# Examination Questions—Royal College of Surgeons of England

**Published:** 1887-12

**Authors:** 


EXAMINATION QUESTIONS.—*Royal College of Surgeons of England*.—The following written questions were given at the Examination held on October 31st.

ANATOMY AND PHYSIOLOGY.

1. Describe the Muciparous Glands lying beneath the front part of the Tongue. Adduce the grounds on which they have been regarded as the seat of Ranula.

2. Describe the hard and soft Palate, the action of their muscles, with the nerve- and blood supply, and give their bearings upon the Surgery of the Palate.

SURGERY AND PATHOLOGY.

3. Give the causes and treatment of Bleeding from the Gums and Jaws.

4. Give the signs and treatment of a Foreign body impacted in the Rima Glottidis.

DENTAL ANATOMY AND PHYSIOLOGY.

1. Describe the dentition and the rostrum of a Saw-Fish. What light is thrown upon the homologies of teeth in general by the teeth of Sharks and Rays?

2. Describe briefly the growth of the Lower Jaw before and after its union at the symphysis. How has its method of growth been traced?

3. Describe minutely the relations of the Upper to the Lower row of Teeth when closed, and point out the advantages of such an arrangement?

DENTAL SURGERY AND PATHOLOGY.

1. What influences are known to injuriously affect Teeth during their development, and how are they subsequently manifested?

2. Give the causes of Alveolar Periostitis. Describe its varieties, treatment, and terminations.

3. What are the difficulties to be encountered in renderiug Stoppings water-tight? How are these to be combated in the application of the several filling-materials in ordinary use.—*Dental Record.*

